# Autobiographical memory after electroconvulsive therapy: systematic review and meta-analysis

**DOI:** 10.1192/bjp.2025.2

**Published:** 2026-03

**Authors:** André Beyer Mathiassen, Maria Semkovska, Christoffer Cramer Lundsgaard, Krzysztof Gbyl, Poul Videbech

**Affiliations:** Center for Neuropsychiatric Depression Research, Mental Health Center Glostrup, Copenhagen University Hospital – Mental Health Services CPH, Glostrup, Denmark; Department of Clinical Medicine, University of Copenhagen, Copenhagen, Denmark; DeFREE Research Unit, Department of Psychology, University of Southern Denmark, Odense, Denmark

**Keywords:** Autobiographical, amnesia, depression, electroconvulsive therapy (ECT), memory

## Abstract

**Background:**

Electroconvulsive therapy (ECT) is the most effective treatment of major depression, but autobiographical memory loss may limit its use. Despite previous attempts to synthesise the literature, the nature of autobiographical memory loss after ECT is still debated.

**Aims:**

To provide an overview of the effect of ECT on autobiographical memory in patients with depression and explore whether the effect is temporary or permanent. Furthermore, we wanted to analyse if ECT parameters or clinical information are associated with this effect.

**Method:**

PubMed, EMBASE, PsycINFO and Web of Science databases were searched on 26 January 2024. We included longitudinal studies measuring autobiographical memory before and after ECT in patients with depression compared to patients with depression receiving other treatment or healthy controls. Synthesis approach was a meta-analysis. PROSPERO ID: CRD42021267901.

**Results:**

Nine studies were included (432 patients, 173 controls). At post-ECT, we found that ECT patients had larger autobiographical memory loss than controls (standardised mean difference (SMD) = 0.55; 95% CI = 0.35–0.75). Right unilateral (RUL) ECT entailed a small effect on autobiographical memory (SMD = 0.32; 95% CI = 0.06–0.57), while bilateral ECT yielded a large effect (SMD = 0.82; 95% CI = 0.49–1.15). Higher age was associated with smaller effect. Autobiographical memory was stable at long-term follow-up.

**Conclusions:**

The studies suggest that ECT causes autobiographical memory loss in patients with depression. Results also suggest that lost memories are not regained. Furthermore, results support that RUL ECT is less detrimental to autobiographical memory. Strangely, a higher age might mitigate the autobiographical memory loss. Our findings are limited by studies being mainly observational and generally consisting of small sample sizes. Future studies should prioritise long-term follow-up assessments of autobiographical memory.

Electroconvulsive therapy (ECT) is the most effective treatment of major depression,^1^ and despite the application of several different types of neuroimaging and blood tests, there is no evidence substantiating that the treatment can damage the brain.^2^ Furthermore, the use of ECT does not increase the risk of later dementia.^3^ However, the cognitive adverse effects may constitute a key limitation to its use.^4^

Some patients report memory loss from events that occurred both during and before an ECT series.^5,6^ A meta-analysis concerning the objective cognitive deficits after ECT found temporary deficits in processing speed, verbal and visual anterograde memory and executive functioning 0–3 days after ECT, resolving within the following 2 weeks.^7^ Loss of personal memories is the adverse effect of greatest concern to patients.^5^ A systematic review suggests that loss of such autobiographical memories does occur after ECT, but primarily concerns relatively recent episodic memories.^8^ However, the exact nature, extent and permanency of the autobiographical memory loss after ECT is still debated,^9–12^ primarily because of methodological problems concerning the validity of the tests used to assess autobiographical memory, the influence of the passage of time and ECT parameters. In addition, depressive episodes per se can cause autobiographical memory deficits.^13,14^ Therefore, it is crucial to control for the effects of such episodes when investigating autobiographical memory deficits in this patient population.^13–17^ To do this optimally, we have decided to focus on the following clinician-rated gold-standard measures: the Hamilton Depression Rating Scale (HAM-D) and the Montgomery–Åsberg Depression Rating Scale (MADRS).

As regards outcome measures for assessing autobiographical memory, it is problematic that several studies have used measures with questionable validity. For example, interviews about public events, news or television programmes entail an innate questionable construct validity, and tools constructed by the authors themselves in relation to single studies also entail questionable validity as well as reliability (e.g. O’Connor et al^18^ and Squire et al^19^). An exception from this is the Kopelman Autobiographical Memory Interview (Kopelman AMI),^20^ which is a validated interview, considered a canonical instrument for the purpose of assessing autobiographical memory in the field of neuropsychology.^21^ The Columbia Autobiographical Memory Interview (CAMI) or -Short Form (CAMI-SF)^22^ is the most frequently used outcome measure for assessing autobiographical memory deficits in the ECT literature.^23^ While the instrument is undoubtedly the most important outcome measure in the field, the validity of the instrument has been debated.^9–12^ Yet, we argue that the instrument possesses a reasonable construct validity. In addition, the inclusion of proper control groups should solve the most important issue of quantifying the amount of autobiographical memory loss by controlling for the effect of time and depression. Therefore, we have decided to focus on these two outcome measures in the present study.

To overcome the above-mentioned methodological challenges, we conducted the present systematic review and meta-analysis. We focus on longitudinal studies, which have used the outcome measures with the highest validity or frequency in the literature, as beforementioned. We include studies with a control group of patients with unipolar or bipolar depression not receiving ECT and studies with healthy control groups to control for both the effect of depression and the passage of time on autobiographical memory, respectively.

First, we aim to provide an updated overview of the effect of ECT on autobiographical memory in patients with depression, including an assessment of the methodological quality of the included studies. Second, we want to explore whether the effect of ECT on autobiographical memory is temporary or permanent. Finally, we want to analyse whether ECT parameters can explain some of the deterioration of autobiographical memory, and if clinical information is associated with such an effect.

## Methods

This review has been conducted following PRISMA guidance^24^ for systematic reviews and meta-analyses and was registered on PROSPERO before data extraction (ID: CRD42021267901). Since registration, changes were made in terms of elaboration of the inclusion criteria and the applied method for quality assessment, and minor changes in the author group. Apart from a post hoc calculation of the number needed to harm (NNH), no changes were made after data extraction.

### Search strategy and selection criteria

We searched for studies in PubMed, EMBASE, PsycINFO and Web of Science databases with no restrictions to language, type of study or publication year. The following search string was used: (ECT OR ‘electroconvulsive therapy’ OR ‘electroshock therapy’ OR ‘convulsive therapy’) AND (autobiographical OR retrograde) AND (memory OR amnesia). The first search was conducted on 8 September 2022 and the last search was performed on 26 January 2024. On this date, ‘OR remote’ was added to the second parenthesis of the search string, but this did not yield any additional relevant studies in any of the databases. The inclusion and exclusion criteria are presented in Table 1. Reference lists of obtained articles were scrutinised for studies not found in the electronic databases.

**Table 1:** Inclusion and exclusion criteria

Inclusion criteria	Exclusion criteria
– Papers written in English – Prospective or retrospective longitudinal studies with measurement of autobiographical memory, as well as antidepressant effect, before and after ECT. – The intervention must be a series of ECT sessions (≥6 sessions). – The intervention group must consist of at least five participants. – The intervention group must consist of human adults (≥18 years old) diagnosed with unipolar or bipolar moderate to severe depression according to DSM-III^30^ or later versions, ICD-8^31^ or later versions or similar (i.e. primary depression research diagnostic criteria). – A control group included, consisting of either healthy controls or patients with unipolar or bipolar depression receiving treatment other than ECT. – The outcome measure for autobiographical memory function must be either Columbia Autobiographical Memory Interview (CAMI), the short form of this instrument (CAMI-SF) or Kopelman Autobiographical Memory Interview (Kopelman AMI). – The outcome measure for antidepressant effect must be Hamilton Depression Ration Scale (HAM-D) or Montgomery–Åsberg Depression Rating Scale (MADRS). – ECT pulse: brief pulse or ultra-brief pulse (UBP), independent of electrode placement.	– Animal studies – Grey literature – Comments or letters to editors – Conference abstracts – Maintenance ECT

ECT, electroconvulsive therapy.

### Data extraction and recorded variables

#### Data extraction

The search results in the electronic databases were checked for duplicates and then screened by reading the titles and/or abstracts by one reviewer. Duplicates were removed automatically using Covidence.org.^25^ Full texts of the chosen articles were retrieved and assessed for inclusion by applying the eligibility criteria (Table 1). The full-text screening was performed by two independent reviewers. Any disagreements were resolved by an additional reviewer. Data extraction was commenced on 28 March 2023, and was also undertaken by two independent reviewers. Any disagreements were resolved by an additional reviewer. If information was missing in any of the papers, the corresponding authors of the papers in question were contacted to obtain additional information.

#### Recorded variables

Extracted variables from all included studies are shown in Table 2. The main outcome measure was the autobiographical memory score. The following categorical variables were extracted with the purpose of examining their potentially moderating effect on this score: type of autobiographical memory test administered, type of control group, type of control intervention, type of study design, blinding of autobiographical memory assessment and electrode placement. Furthermore, the following continuous variables were extracted with the same purpose: age, the percentage of patients with unipolar depression, medication statuses in percentages, pre-ECT depression severity score, pre–post change in this score and delay of post-ECT autobiographical memory assessment, as well as the remaining ECT parameters (see Table 2).

**Table 2:** Overview of the extracted variables

Subject characteristics	Study design	ECT parameters	Depression outcome	Autobiographical memory outcome
Number of patients in ECT and control groups Gender (% female) Age (mean, s.d.) Percentage unipolar depression Washout period (yes/no) Medication status (percentage of antidepressant treatment, antipsychotic treatment and mood stabilising treatment, respectively)	Type of study design (e.g. RCT, observational) Type of control group (depression, healthy control) Type of control intervention (medication, other non-ECT, none) Blinded autobiographical memory assessment (yes, no)	Electrode placement (RUL, bilateral, mixed) Pulse width (brief pulse, UBP and specific ms) The number of ECT treatments per week The number of ECT sessions in total (mean, s.d.) Type of ECT machine (e.g. MECTA, Thymatron and versions) Treatment dose (mC; mean, s.d.) Duration of EEG seizures (seconds; mean, s.d.)	Type of test administered (MADRS, HAM-D, and versions) Baseline/pre-ECT depression severity score (mean, s.d.) Immediate follow-up/post-ECT depression severity score (mean, s.d.)	Type of test administered (CAMI, CAMI-SF, Kopelman AMI) Baseline/pre-ECT autobiographical memory score (mean, s.d.) Immediate follow-up/post-ECT autobiographical memory score (mean, s.d.) Long-term follow-up autobiographical memory score (mean, s.d.) Delay of post-ECT assessment as well as later follow-up assessments (days after last ECT session)

ECT, electroconvulsive therapy; RCT, randomised controlled trial; RUL, right unilateral; MADRS, Montgomery–Åsberg Depression Rating Scale; HAM-D, Hamilton Depression Rating Scale; CAMI, Columbia Autobiographical Memory Interview; CAMI-SF, Columbia Autobiographical Memory Interview-Short Form; Kopelman AMI, Kopelman Autobiographical Memory Interview; UBP, ultra-brief pulse; EEG, Electroencephalography.

Delay of post-ECT assessment, as well as later follow-up assessments, was coded in days, and where relevant transformations from months to days were calculated (1 month = 30.42 days). Depression severity scores were transformed to the HAM-D using standard transformation formulae.^26^

### Data analysis

#### Meta-analysis

The effect sizes for the main outcome from each study were expressed as the standardised mean difference (SMD) between the pre–post-ECT change in autobiographical memory observed in the ECT group and in the control group. First, Cohen’s *d* index of individual effects was calculated for each individual sample (either ECT or control) from each study using the following:

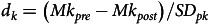

where *d* is the effect size, *k* is the individual sample, *Mk*_*pre*_ is the pre-treatment (or baseline) mean on the autobiographical memory measure, *Mk*_*post*_ is the post-treatment (or retest) mean on the autobiographical memory measure and *SD*_*pk*_ is the pooled s.d. Then, the SMD was computed as the difference between the *d*_*k*_ of the ECT group and the *d*_*k*_ of the control group. The SMD was adjusted for small sample size bias using Hedges’ *g*,^27^ and interpreted according to Cohen’s^28^ recommended cut-offs of 0.80, 0.50 and 0.20 for large, moderate and small effect sizes, respectively. All meta-analyses were conducted with Comprehensive Meta-Analysis version 2.2 for Windows (Biostat, Englewood, NJ, USA; see http://meta-analysis.com).

For studies with more than two test time points (i.e. more than one post-treatment assessment), the synthetic variable Hedges’ *g* correction^29^ was applied to avoid those studies being unduly weighted because of multiple retest time points. Adjusted between-group SMDs were pooled using random-effect models, as significant heterogeneity was expected. To determine if the pooled SMD was unduly influenced by single studies, a sensitivity analysis was conducted, recomputing the pooled SMD after deleting each between-group SMD from individual studies, one at a time.

Homogeneity of the adjusted SMD was assessed with the *I* ^2^ statistic^29^, interpreted according to the recommended cut-offs of 25%, 50% and 75% representing low, moderate and high heterogeneity, respectively.^29^ When significant heterogeneity was found (*I* ^2^ ≥ 25%), we investigated the pre-specified moderators’ capacity to explain variability. For categorical moderators (type of autobiographical memory test administered, type of control group, type of control intervention, type of study design, blinding of autobiographical memory assessment and electrode placement), subgroup mixed-effects analyses were used when at least two studies were available per moderator level.^32^ This mixed-effects model assumes that the pre-specified characteristics act as moderator variables and partly explain the variability among effect sizes, while also allowing for a random component of residual variance to remain after accounting for the moderator portions. For the remaining continuous moderators (e.g. mean age (ECT group) and the delay of post-ECT autobiographical memory assessment), the random-effects model, method of moments and meta-regressions were conducted when ≥10 samples contained data on the corresponding moderator variable.

#### Post hoc analysis: number needed to harm

The NNH was calculated by use of ClinCalc^33^ and the analysis was based on the comprehensive data-set provided by Sackeim et al.^34^ As raw data were not available for the healthy control group, we compared the bilateral to the right unilateral (RUL) electrode placement in the NNH analysis.

The cut-off for adverse autobiographical memory loss (AAML) was set to two s.d. below the mean score of the applied control group, since this is a conservative standardised cut-off for cognitive impairment.^21^ Thus, the resulting cut-off for AAML was below 60.6% consistency at immediate follow-up. Because of the lack of available normative data, the control group of the included randomised controlled trial (RCT),^35^ which consisted of patients with bipolar depression receiving pharmacotherapy (*n* = 20), was applied. They were assessed with the CAMI-SF at baseline and a mean of 54.0 (s.d. = 16.9) days thereafter. At the latter time point, the depression group had a mean of 80.8% (s.d. = 10.1) consistency on the CAMI-SF. The variance of the consistency at 6 months follow-up was not stated in the follow-up study,^36^ and therefore the long-term NNH could not be calculated.

#### Risk of bias assessment and methodological quality

Publication bias was assessed with Egger’s test of significance of bias. For significant results, a fail-safe *N* file-drawer analysis^37^ was also conducted to determine how many unpublished studies with null findings would negate the significant findings derived from published studies.

The risk of bias and the methodological quality were assessed for all included studies. The Revised Cochrane risk of bias 2 (RoB 2) tool^38^ was used for RCTs. The Newcastle–Ottawa scale (NOS)^39^ was used for non-randomised studies, and evaluations were interpreted according to guidelines.^40^ The evaluations were conducted by two reviewers, and any disagreements were solved through consensus.

### Ethics statement

As we did not collect data from individual participants, but only extracted data from already published papers for the purpose of synthesis, formal ethical approval was not deemed relevant.

## Results

**Fig. 1:**
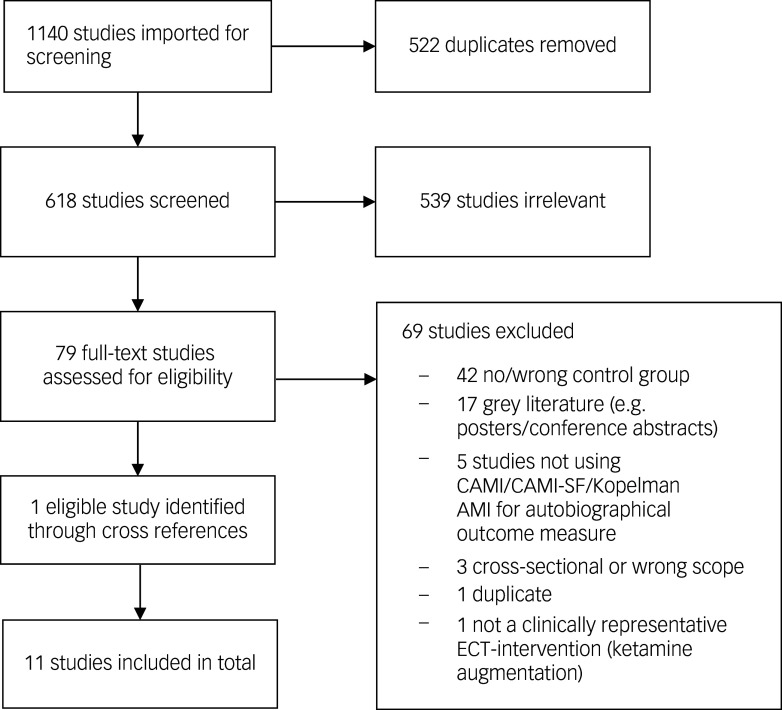
Flowchart of the study selection process. CAMI, Columbia Autobiographical Memory Interview; CAMI-SF, Columbia Autobiographical Memory Interview-Short Form; Kopelman AMI, Kopelman Autobiographical Memory Interview; ECT, electroconvulsive therapy.

A flowchart of the study selection process is depicted in Fig. 1.

The corresponding authors of all the studies were contacted via email with a request for additional data, aiming at a complete data-set. Of all the studies, five authors returned with the requested data wholly or partly because of varying availability. The remaining authors either did not respond within a reasonable time or responded that data was no longer available. In two cases, the authors did not provide the necessary raw data to be included in either the meta-analysis or even the narrative review of our research question.^41,42^ Therefore, nine studies in total were an effective part of the review and meta-analysis. The characteristics of each of these studies are presented in Table 3. Regarding the study by Sackeim et al,^34^ raw data were received, of which we removed all patients with a diagnosis of schizoaffective disorder and all patients who received sine-wave ECT. Thus, only the data of the remaining participants were included in the meta-analysis.

**Table 3:** Overview of included studies

		ECT group					Control group						
Author	Design	Electrode placement	Pulse width (ms)	*n* ^a^	Age^b^, mean (s.d.)	Gender, % female	Diagnosis	Intervention	*n* ^a^	Age^b^, mean (s.d.)	Gender, % female	Outcome measure	Results (percentage consistency in autobiographical memory outcome)
Bjoerke-Bertheussen et al (2018)^36^	RCT	RUL	0.5	15	46.5 (10.8)	66.7	Depression	Pharma	11	42.9 (11.5)	72.7	CAMI-SF	ECT 6 mo.: 64.7% Pharma 6 mo.: 65.3% Not significant.
Kessler et al (2014)^35^	RCT	RUL	0.5	19	46.0 (10.2)	57.9	Depression	Pharma	20	42.9 (11.2)	60.0	CAMI-SF	ECT post: 73.0% Pharma post: 80.7% A significant interaction between treatment group and time: η^2^ = 0.14.
Semkovska and O’Grady (2017)^43^	Obs.	Bitemporal	0.5	19	47.8 (12.3)	52.6	Depression	Pharma	19	47.2 (12.0)	52.6	CAMI-SF (Semkovska et al scoring system^23^)	ECT post: 62.6% Pharma post: 80.2% Significant difference. ECT 3 mo.: 57.8% Pharma 3 mo.: 74.5% Significant difference.
Bergfeld et al (2017)^44^	Obs.	RUL/bitemporal	0.5	10	45.4 (9.0)	71.4	Depression Healthy controls	DBS Time	18	53.2 (8.4)	68.0	CAMI-SF (Semkovska et al scoring system^23^)	ECT post: 45.8% DBS: n.a. Healthy controls: 84.0% Not tested for significance. ECT 1 yr.: 49.0% DBS 1 yr.: 60.9% Healthy controls 1 yr.: 79.5%
									20	53.5 (8.0)	61.9		
Blomberg et al (2020)^45^	Obs.	RUL	0.5	23	45.3 (14.6)	52.6	Healthy controls	Time	15	40.6 (16.2)	62.6	CAMI-SF	ECT 6 mo.: 70.30% Healthy controls 6 mo.: 82.03% *p* = .0005
Dybedal et al (2014)^46^	Q.exp.	RUL/bifrontal	0.5	62	74.7 (6.6)	53.2	Healthy controls	Time	17	78.1 (4.5)	64.7	CAMI-SF (modified)	ECT post: 90% Healthy controls post: 90% No difference.
Sackeim et al (2007)^34^	Obs.	RUL/bilateral	N/A	265	56.7 (17.6)	63.1	Healthy controls	Time	24	54.8 (19.2)	N/A	CAMI-SF	ECT post: 60.9% Healthy controls post: n.a. ECT 6 mo.: 60.7%
Schulze-Rauschenbach et al (2005)^47^	Obs.	RUL	N/A	14	46.7 (11.0)	50.0	Depression Healthy controls	rTMS Time	16	47.7 (13.1)	43.8	Kopelman AMI (shortened version)	ECT post: 98.9% rTMS post: 102.7% Healthy controls post: 102% Not tested for significance.
									15	48.9 (13.8)	46.7		
Weeks et al (2013)^48^	Q.exp.	Bifrontal	N/A	20	41.6 (12.5)	40.0	Depression	Isoflurane anaesthesia	8	35.4 (9.9)	62.5	CAMI-SF	ECT post: 69.6% ISO post: 92.7% Significant difference. ECT 1 mo.: 69.6% ISO 1 mo.: 89.5% Significant difference.

ECT, electroconvulsive therapy; percentage consistency, the proportion of answers matching the answers given at baseline; RCT, randomised controlled trial; RUL, right unilateral; Pharma, pharmacotherapy; CAMI-SF, Columbia Autobiographical Memory Interview-Short Form; DBS, deep brain stimulation; Q.exp, quasi-experimental; N/A, not available; Obs., observational; rTMS, repetitive transcranial magnetic stimulation; Kopelman AMI, Kopelman Autobiographical Memory Interview; ISO, isoflurane anaesthesia.

a Number of completers.

b Age in years.

### Meta-analysis

**Fig. 2:**
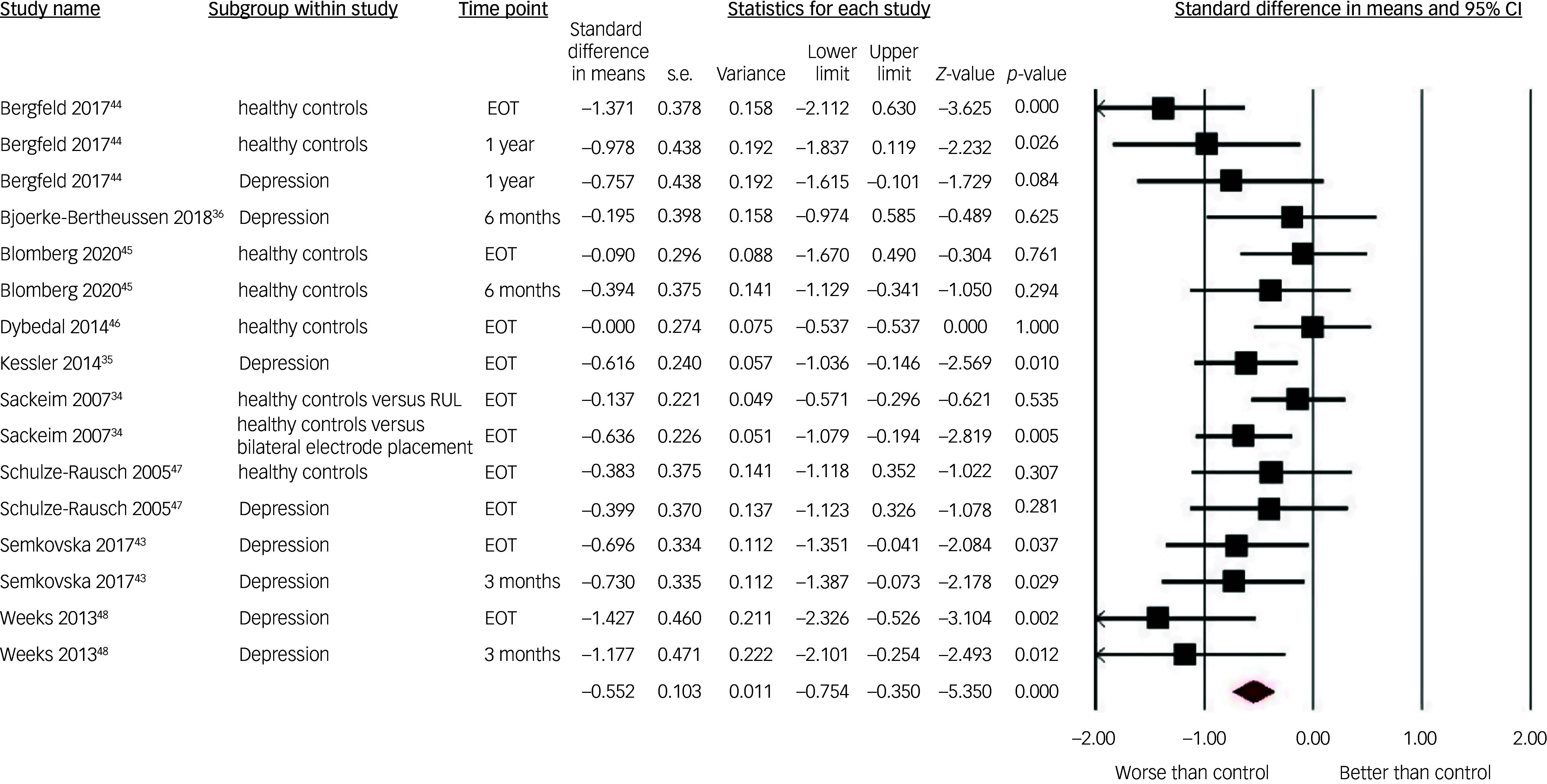
Forest plot of effect sizes of electroconvulsive therapy on autobiographical memory in each included study by subgroup. RUL, right unilateral electrode placement; EOT, end of treatment.

The adjusted pooled SMD showed moderate superiority of the pre–post-ECT change in autobiographical memory in the controls relative to the ECT patients: 0.55 (95% CI 0.35–0.77) with a low level of heterogeneity *I* ^2^ = 35.11 (see Fig. 2). Sensitivity analyses showed robust results, with no outliers appearing with one study removed: pooled SMD remained between 0.51 (Weeks et al^48^ removed) and 0.60 (Sackeim et al^34^ removed), thus close to the adjusted mean and within its 95% confidence interval.

See Tables 4 and 5 for the results of the pre-specified moderator analyses. Two significant moderators of autobiographical memory SMD were found. For electrode placement, a large effect size between patients treated with bilateral ECT and controls was found. This was significantly different from the small effect size observed when comparing patients treated with RUL ECT to controls. Increased patients’ age was associated with smaller autobiographical memory SMD between the ECT and the matched control groups. All other pre-specified moderators did not significantly affect the differences in pre–post change in autobiographical memory between patients treated with ECT and their controls. Notably, this included the type of control group (healthy controls/patients with depression). Therefore, it was not meaningful to perform separate analyses of ECT versus healthy controls and ECT versus patients with depression, since the effect sizes did not differ with the type of control group. Accordingly, all SMDs were pooled, regardless of the type of control group applied in the different studies.

**Table 4:** Categorical moderators’ effects on the standardised mean difference between the pre–post-electroconvulsive therapy (ECT) change in autobiographical memory consistency observed in the ECT group and control groups

Categorical moderators	*k*	SMD	95% CI	*Qb*	*P*
Control group					
Healthy controls	7	0.44	0.14–0.75		
Uni-/bipolar depression treated with antidepressants	6	0.76	0.39–1.11	1.75	0.42
Design					
Observational	11	0.56	0.29–0.82		
Quasi-experimental	3	0.67	0.13–1.22		
RCT	2	0.46	0.13–1.05	0.27	0.87
Blinding					
No	12	0.60	0.36–0.84		
Yes	4	0.43	0.02–0.85	0.45	0.50
Electrode placement					
Bitemporal	5	0.82	0.49–1.15		
Mixed	4	0.64	0.25–1.03		
Right unilateral	7	0.32	0.06–0.57	6.05	0.049
Bitemporal versus mixed	9	_–_	_–_	0.24	0.62
Bitemporal versus right unilateral	12	_–_	_–_	6.81	0.009
Mixed versus right unilateral	11	_–_	_–_	1.83	0.18

*k*, number of patient samples; SMD, standardised mean difference; *Qb*, between-group heterogeneity; RCT, randomised controlled trial; Mixed, a mixed sample of participants who received either right unilateral ECT or bilateral ECT, or were switched from one to the other.

**Table 5:** Continuous moderators’ effects on the standardised mean difference between the pre–post-electroconvulsive therapy (ECT) change in autobiographical memory observed in the ECT group and control groups

Continuous moderators	*k*	*z*	*P*
Interval (days)	16	−0.97	0.33
Age (years)	11	1.98	0.048
Gender (% women)	11	0.40	0.69
Number of ECT sessions	11	0.86	0.39
Mean improvement in depression^a^	9	−0.80	0.42

*k* = number of patient samples; *P* = significance level; *z* = test of significance of the regression slope.

a Analysis performed with lower than the minimum *k* =10 required.

The effect of the following pre-specified moderators could not be assessed: autobiographical memory test used, pulse width and % of patients diagnosed with unipolar depression. The reasons for this were either insufficient data to perform a meta-regression (% of patients diagnosed with unipolar depression) or lack of variability in moderator levels, that is, only one study^47^ used the Kopelman AMI, whereas all remaining studies used the CAMI or CAMI-SF, and all studies used brief-pulse ECT.

As only four studies performed a long-term follow-up assessment, here defined as 6 months after treatment completion^34,36,45^ or later (1 year in the case of the last study^44^), it was not possible to conduct a meta-analysis of the long-term effects. However, the studies were coherent as they all showed lower or stable mean autobiographical memory consistency at the long-term follow-up compared to the immediate follow-up assessment. In addition, they all showed numerically lower autobiographical memory consistency after 6 months/1 year compared to their control groups. In two cases, this difference did not reach statistical significance.^36,44^

### Risk of bias assessment

**Fig. 3:**
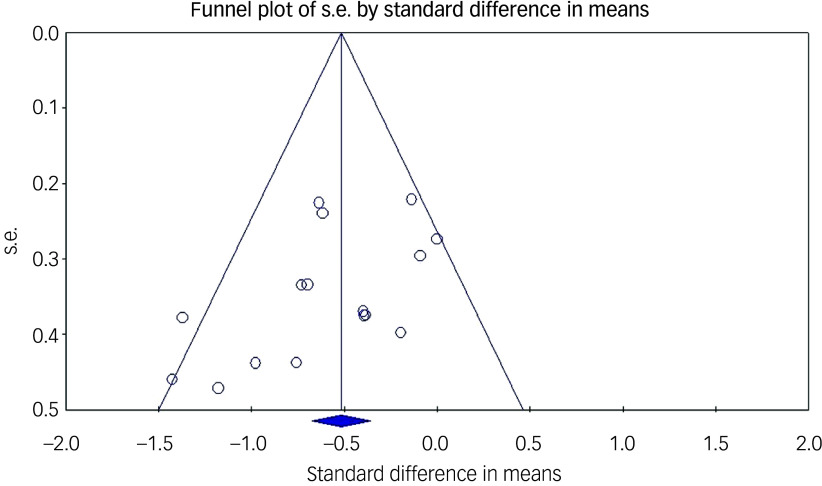
Funnel plot of publication bias.

The Egger’s regression intercept test was significant (*t* = 2.22, *P* = 0.022), suggesting the presence of a publication bias in reporting the main outcome. Inspection of the corresponding funnel plot showed evidence for publication bias favouring publication of larger differences between the ECT and control groups (see Fig. 3). However, fail-safe analyses showed the need for 180 unpublished studies with nil results to negate the significance of the main outcome, suggesting that the real effect might, despite the publication bias, still be significant, but possibly of a smaller size.

The overall assessment of risk of bias using the RoB 2 tool indicated a high risk of bias in both RCTs analysing the adverse effects of ECT versus antidepressant medication. For more details, see Table 6.

**Table 6:** Risk of bias assessment of randomised controlled trials using RoB 2

Study	Randomisation process	Deviation from intended intervention	Missing outcome data	Measurement of the outcome	Selective reporting	Overall
Bjoerke-Bertheussen et al (2018)^36^	Low	High	High	Low	Some concerns	High
Kessler et al (2014)^35^	Low	Low	Some concerns	High	Low	High

RoB 2, Revised Cochrane risk-of-bias tool for randomised trials.

The overall methodological quality of the seven non-randomised studies was assessed to be good according to guidelines,^40^ based on a mean score of 7.7 out of 9 possible stars (more stars indicating better methodological quality) on the NOS and a quite homogeneous scoring between studies. For more details, see Table 7.

**Table 7:** Methodological quality assessment of non-randomized studies using NOS

Study	Selection	Comparability	Outcome
Bergfeld et al (2017)^44^	****	**	***
Blomberg et al (2020)^45^	***	*	**
Dybedal et al (2014)^46^	***	*	**
Sackeim et al (2007)^34^	***	*	**
Schulze-Rauschenbach et al (2005)^47^	****	**	**
Semkovska et al (2017)^43^	****	**	***
Weeks et al (2013)^48^	****	**	***

NOS, Newcastle–Ottawa Scale. Selection, Comparability and Outcome can be granted a maximum of 4, 2 and 3 stars, respectively. More stars indicate better methodological quality.

### Post hoc analysis: number needed to harm

In the RUL group (*n* = 139) 36.0% had AAML immediately after ECT, while 54.8% had AAML in the bilateral group (*n* = 126). The NNH analysis showed that five patients would have to receive bilateral instead of RUL ECT for one additional patient to have AAML.

## Discussion

Overall, we found a moderate effect of ECT on autobiographical memory compared to control groups, with a low level of heterogeneity between the included studies. Furthermore, we found a moderating effect of electrode placement, with bilateral placement showing a large effect on autobiographical memory, while RUL placement only showed a small effect on autobiographical memory. The difference in the effect of bilateral versus RUL electrode placement on autobiographical memory can be more tangibly understood from our calculation of the NNH of 5. This means that approximately five patients would have to receive bilateral instead of RUL ECT for one additional patient to have AAML. We also found a higher age to be associated with a smaller effect on autobiographical memory. The amount of autobiographical memory loss remained stable between end-of-treatment and long-term follow-up (6–12 months), indicating that autobiographical memory consistency does not improve with the passage of time following ECT.

Our results are in accordance with previous findings that ECT causes autobiographical memory loss,^8^ as well as studies finding a greater autobiographical memory loss/retrograde amnesia after bilateral compared to RUL ECT.^49–53^ Regarding the moderating effect of age, our result is in line with an earlier finding that a higher age is associated with less autobiographical memory loss after ECT.^54^ In contrast, another study found the opposite association,^41^ and others did not find any association between age and autobiographical memory loss after ECT.^34,55–57^ We cannot rule out that this correlation with age is caused by a better antidepressant effect in older patients,^58^ but we did not have sufficient statistical power to explore this. In addition, a possibly inferior baseline memory performance by older compared to younger patients could constitute a floor effect in detecting autobiographical memory loss after ECT in older patients.

### ECT versus healthy controls

The results of the five studies comparing the effect of ECT on autobiographical memory with healthy controls are consistent, as all^34,44,45,47^ but one study^46^ found larger decreases in autobiographical memory consistency in the ECT group immediately after ECT. In addition, three of the studies^34,44,45^ found a difference in favour of the healthy control groups at long-term follow-up (6–12 months).

One study^46^ used a modified version of the CAMI-SF, seemingly limiting the sensitivity to recent episodic memory loss, as the two most sensitive items for this purpose (Last Birthday and Last New Year’s Eve) had been removed. Although they included Last Christmas Eve as a substitute item, one might argue that it encourages even more semantic answers. This, and the fact that the interval between baseline and follow-up assessment was longer for the healthy controls (M = 58.2 days; s.d. = 4.4) than for the ECT group (M = 37.5; s.d. = 10.6), could possibly explain why the authors did not find a separable effect of ECT on autobiographical memory compared to healthy controls.

There is variability in the degree of autobiographical memory deterioration observed by the different studies. This could be because of the use of the Kopelman AMI as an outcome measure,^47^ which is an interview emphasising semantic retrograde memory to a larger degree than the CAMI-SF, and thus is less sensitive to recent episodic memories. The use of a low electrical dose of the RUL ECT (2–2.5 × the seizure threshold) could also be a factor in one of the studies,^47^ as it is known to be a relatively ineffective dose for unilateral electrode placement.^59^ A discrepancy between a significantly larger decrease in autobiographical memory consistency at long-term follow-up in the ECT group versus healthy controls but not at immediate follow-up (although also numerically larger)^45^ could be because of the follow-up visit taking place 19 days after the last ECT, thus ameliorating acute effects. It could also partly be an effect of depression and should either way be interpreted with caution. One might also speculate that anterograde memory difficulties in the ECT group could contribute to the difference at 6 months, as it would limit the practice effects of repeating the memories at T1 and T2, which opposingly is benefitting the healthy controls group.

### ECT versus non-ECT in patients with depression

#### ECT versus psychopharmacological treatment

As depression is expected to have a deteriorating effect on autobiographical memory^13,14^ it is crucial to not only control for the passage of time but also for the effect of depression. The three studies comparing the effect of ECT on autobiographical memory with patients with depression receiving pharmacotherapy were based on two samples, as one study^36^ was the long-term follow-up of another.^35^ Both study samples showed a significantly lower autobiographical memory consistency after ECT at the short-term follow-up assessment compared to their control group.^35,43^ One study found a significantly lower autobiographical memory consistency in the ECT group after 3 months,^43^ while the other failed to find a significant difference after 6 months,^36^ although there was still a numerical difference in consistency (64.3% and 72.3%, favouring the control group).

The nil-finding in the first-mentioned study at 6 months follow-up^36^ can possibly be explained by several factors. Nearly half of the sample dropped out before the 6-month assessment, leaving only 11 and 15 in the pharmacotherapy and ECT groups, respectively, which might not yield sufficient power to reach statistical significance. RUL electrode placement was applied as opposed to the other study using bitemporal electrode placement, the latter being known to cause more pronounced cognitive deficits.^51^ The authors used a pulse width of 0.5 ms^35,36^ as opposed to 1.0 ms in the other study.^43^ In terms of outcome, they analysed the sum of the total scale, while the other study only demonstrated a significant difference on the most sensitive items of the instrument, Episodic-Specific,^43^ as defined by Semkovska et al.^23^ A clear strength, however, was the blinded RCT design^35,36^ in contrast to the retrospective case–control design applied in the other study.^43^ The latter study did match the ECT group to a control group with a similar level of depression but confounding by indication, presumably including treatment resistance, cannot be ruled out. In addition, they applied an alternative scoring system, which further limits the direct comparability of the studies.

#### ECT versus non-psychopharmacological treatment

The three studies comparing the effect of ECT on autobiographical memory with patients with depression receiving other antidepressive treatments all found a numerically larger decrease in autobiographical memory after ECT compared to their control group,^44,47,48^ although only two of three studies found the difference to be statistically significant within the first week after the last ECT^44,48^ and at 1-month follow-up in the latter study.^48^ The other study observed a nil-finding at long-term follow-up of 60.3 weeks (s.d. = 29.5) after ECT, but the comparison is considered invalid, as the control group was assessed 122.7 weeks (s.d. = 22.2) from baseline.^44^

The discrepancies in the studies could be caused by the control group interventions being heterogeneous (see Table 3), and their cognitive side-effects seeming rather unexplored. The study that did not find the difference in autobiographical memory consistency decline to be statistically significant applied RUL electrode placement,^47^ as opposed to the beforementioned study applying bilateral electrode placement.^48^ Application of an ineffective electrical dose,^47^ reflected in a low response rate in the ECT group of 46%,^47^ might also have hindered a statistically significant difference from being detected. Usage of the Kopelman AMI,^47^ probably limiting the sensitivity to change, might elicit the same effect. Differences in baseline HAM-D scores could also have affected the results in one of the studies.^48^ As all three studies were observational in design, group differences caused by confounding by indication cannot be ruled out. In addition, the mean sample sizes of these three studies are smaller than that of the studies in the two preceding sections, thus serving as an important limitation.

### Negative findings

Contrary to our expectations we found no moderating effect of the type of control group on the effect of ECT on autobiographical memory. Although the depression control groups in general were very similar to the ECT groups in terms of baseline depression severity, most study designs were observational, and patients were therefore not randomised to the treatments. This heightens the possibility that there is a confounding by indication effect in these studies, as the patients in the depression control groups probably were less ill than the ECT patients. This might limit the deteriorating effect that the depressive episode has on their autobiographical memory recall, thus approaching similarity with the autobiographical memory recall efficiency of the healthy controls. Consequently, this would, to some extent, magnify the effect of ECT on autobiographical memory, inappropriately. Another possible explanation for the lack of moderating effect of the type of control group is that the effect of depression itself on autobiographical memory recall is more subtle, covering a tendency to overgeneralisation and negative bias of the memories^60,61^ rather than pure recall failures per se.

We expected that the number of ECT sessions would moderate the effect of ECT on autobiographical memory, which it did not. This could be because of the fact that the variance in the number of ECT sessions received in the individual studies was quite high in general (mean s.d. = 3.2). The moderator analysis is based on the mean number of ECT sessions received in the individual studies, which thus might not be sufficiently representative of the true variance for the moderating effect to reach statistical significance.

### Psychometric properties and validity of the CAMI/CAMI-SF

While the CAMI/CAMI-SF is the most frequently used tool to assess autobiographical memory loss in the ECT literature, its validity has been debated.^9–12^ Performance at follow-up assessments is scored as the degree of consistency with the baseline score, which has been criticised as it is not possible to achieve a score higher than that of the baseline. The critique is somewhat just, as we cannot know with absolute certainty whether some patients exert more accurate retrieval of autobiographical memories after ECT as opposed to before. However, the literature indicates that the opposite is the case,^8^ not only because of ECT but also the effect of time and depression.^13,14^ Baseline recall might, in some cases, be vague and limited in detail as an effect of depression,^61^ but outright confabulation is unlikely, as this seems to be a rare phenomenon in depression^62^ and, if anything, more likely after ECT.^62^ It has further been proposed that the instrument is not able to reliably determine whether the retrograde amnesia is persistent long term since scores above baseline cannot be attained. This point seems unjustified, as it is possible to improve from the deterioration at immediate follow-up back to the baseline score at later follow-up assessments. The fact that one cannot score higher than baseline does not make the instrument incapable of determining if autobiographical memory loss is persistent or not, even less so since the normal trajectory is to lose consistency over time.

What seems like a more compelling limitation of the CAMI-SF is the probable lack of sensitivity to episodic autobiographical memories, and perhaps one can even question the validity of the episodic items. The six items of the CAMI-SF are predominantly concerned with memories that are more than 1 year old (some can even go decades back) and some of the questions even call for semantic memory recall. We can only be certain that two of the items ask about memories within the past year, that is, Last New Year’s Eve and Last Birthday. However, even this might be too wide a time interval for it to be sensitive enough to the retrograde memory deficits of patients after ECT, as it has earlier been found that the 6 months leading up to ECT is the most affected period.^8^ Furthermore, the questions asked in these two items seem to elicit semantically/schemata supported answers to some degree, since it is not infrequent that people have traditions for their birthdays and New Year’s Eves, entailing perhaps the same location, activities, guests, gifts, foods and beverages. Thus, the CAMI-SF seems to have a reduced sensitivity to the episodic autobiographical memory loss after ECT, more than the opposite. Consequently, one seems mostly prone to committing type II errors regarding the autobiographical memory loss after ECT, using this instrument.

Another important critique of the CAMI/CAMI-SF is the lack of demonstrated validity and reliability. To our knowledge, a formal validation has not been published for the original forms of the instrument, which is an important limitation. Lack of available normative data has further been proposed as a critique. This is a reasonable demand from a clinical perspective, but for scientific studies the inclusion of proper control groups should suffice.

All in all, when evaluating this instrument, we consider it to have a reasonable degree of construct validity yet providing a conservative representation of the autobiographical memory loss following ECT, when contrasted to comparable control groups.

### Strengths and limitations

The most important strength of the present systematic review is the fact that we applied strict criteria for study inclusion. Many studies were excluded since they did not have a non-ECT control group. This is important because a control group like this is necessary to control for the effect of time and depression severity, which are two of the most important confounding variables when attempting to measure the effect of ECT on autobiographical memory in patients with depression. Furthermore, we were strict in only including studies using validated/consensus outcome measures. This heightens the validity of our results and enables us to make a homogeneous synthesis.

An important limitation of our review is the proportions of the different study designs applied in the included studies, as they predominantly are observational, and only one original study is a RCT. That said, the naturalistic designs do have strengths, as they are more clinically representative. Another limitation of our review is the fact that the validity of the most frequently used outcome measure, the CAMI/CAMI-SF, has been a subject of discussion for years; refer to the points outlined in the section ‘Negative findings’, although we argue that the assessment tool is useful yet conservative in its measurement. In addition, the studies, with one exception, consisted of rather small sample sizes; however, this is a known characteristic of the field in general.

## Conclusion

Our systematic review suggests that ECT causes autobiographical memory loss in patients with depression. This is supported both narratively and by our meta-analysis revealing an overall moderate effect of ECT on autobiographical memory compared to controls. The autobiographical memory loss remains stable between end-of-treatment and long-term follow-up (6–12 months), indicating that lost memories are not regained.

Furthermore, our meta-analysis showed that electrode placement significantly moderated the effect of ECT on autobiographical memory. RUL ECT, mixed position and bilateral ECT entailed small, moderate and large effects on autobiographical memory, respectively. Accordingly, the NNH was five when comparing bilateral with RUL ECT. Furthermore, a higher age was associated with a smaller negative effect on autobiographical memory after ECT, but this finding warrants further research.

The present findings are limited by risk of bias in the included studies to some extent. Most importantly, this is because of small sample sizes, treatment parameters and possible selection bias caused by a general lack of randomised designs. Note, it is possible that the moderating effect of RUL placement is slightly underestimated, and the estimation of the long-term autobiographical memory loss should be considered tentative.

High-dose RUL ECT has been shown to be as effective as bilateral ECT as regards antidepressant efficacy, while also resulting in less autobiographical memory loss.^51^ Our results support the latter finding and thus emphasise the importance of considering RUL ECT as a viable treatment alternative to bilateral ECT in the treatment of patients with moderate to severe depression.

Future studies should prioritise conducting long-term follow-up assessments of autobiographical memory in ECT patients compared with control groups with similar depression severity, as this would test our tentative conclusion regarding the permanency of autobiographical memory loss following ECT.

## Supporting information

Mathiassen et al. supplementary material 1 Mathiassen et al. supplementary material

Mathiassen et al. supplementary material 2 Mathiassen et al. supplementary material

## Data Availability

The data that support the findings of this study are available from the corresponding author, A.B.M., upon reasonable request, and to the extent that the corresponding authors of the included studies consent to the distribution of their data.

## Supplementary material

The supplementary material is available online at https://doi.org/10.1192/bjp.2025.2
