# Supplementary material for: Autobiographical memory after electroconvulsive therapy: systematic review and meta-analysis
Source: Br J Psychiatry. 2025 May 13;228(3):263–73. doi: 10.1192/bjp.2025.2 (PMC12916233; doi:10.1192/bjp.2025.2)
Supplement: Mathiassen et al. supplementary material 1 — Mathiassen et al. supplementary material [file S0007125025000029sup001.pdf]

List of all included papers

Bergfeld, I. O., Mantione, M., Hoogendoorn, M. L. C., Horst, F., Notten, P., Schuurman, P. R., & Denys, D. (2017). Episodic memory following deep brain stimulation of the ventral anterior limb of the internal capsule and electroconvulsive therapy. *Brain Stimulation*, *10*(5), 959–966. https://doi.org/10.1016/j.brs.2017.07.006

Bjoerke-Bertheussen, J., Schoeyen, H., Andreassen, O. A., Malt, U. F., Oedegaard, K. J., Morken, G., Sundet, K., Vaaler, A. E., Auestad, B., & Kessler, U. (2018). Right unilateral electroconvulsive therapy does not cause more cognitive impairment than pharmacologic treatment in treatment-resistant bipolar depression: A 6-month randomized controlled trial follow-up study. *Bipolar Disorders*, *20*(6), 531–538. https://doi.org/10.1111/bdi.12594

Blomberg, M. O., Semkovska, M., Kessler, U., Erchinger, V. J., Oedegaard, K. J., Oltedal, L., & Hammar, Å. (2020). A Longitudinal Comparison Between Depressed Patients Receiving Electroconvulsive Therapy and Healthy Controls on Specific Memory Functions. *Primary Care Companion for CNS Disorders*, *22*(3), e1–e7. https://doi.org/10.4088/PCC.19m02547

Dybedal, G. S., Tanum, L., Sundet, K., Gaarden, T. L., & Bjølseth, T. M. (2014). Cognitive side-effects of electroconvulsive therapy in elderly depressed patients. *The Clinical Neuropsychologist*, *28*(7), 1071–1090. https://doi.org/10.1080/13854046.2014.958536

Kessler, U., Schoeyen, H. K., Andreassen, O. A., Eide, G. E., Malt, U. F., Oedegaard, K. J., Morken, G., Sundet, K., & Vaaler, A. E. (2014). The effect of electroconvulsive therapy on neurocognitive function in treatment-resistant bipolar disorder depression. *Journal of Clinical Psychiatry*, *75*(11), e1306–e1313. https://doi.org/10.4088/JCP.13m08960

Sackeim, H. A., Prudic, J., Fuller, R., Keilp, J., Lavori, P. W., & Olfson, M. (2007). The cognitive effects of electroconvulsive therapy in community settings. *Neuropsychopharmacology : Official Publication of the American College of Neuropsychopharmacology*, *32*(1), 244–254. https://doi.org/10.1038/sj.npp.1301180

Schulze-Rauschenbach, S. C., Harms, U., Schlaepfer, T. E., Maier, W., Falkai, P., & Wagner, M. (2005). Distinctive neurocognitive effects of repetitive transcranial magnetic stimulation and electroconvulsive therapy in major depression. *British Journal of Psychiatry*, *186*(MAY), 410–416. https://doi.org/10.1192/bjp.186.5.410

Semkovska, M., & O'Grady, T. (2017). Unravelling Autobiographical Retrograde Amnesia Following Bitemporal Electroconvulsive Therapy: Effect of Treatment versus Effect of Time. *Psychology*, *08*(04), 611–626. https://doi.org/10.4236/psych.2017.84039

Weeks, H. R., Tadler, S. C., Smith, K. W., Iacob, E., Saccoman, M., White, A. T., Landvatter, J. D., Chelune, G. J., Suchy, Y., Clark, E., Cahalan, M. K., Bushnell, L., Sakata, D., Light, A. R., & Light, K. C. (2013). Antidepressant and Neurocognitive Effects of Isoflurane Anesthesia versus Electroconvulsive Therapy in Refractory Depression. *PLoS ONE*, *8*(7), 1–8. https://doi.org/10.1371/journal.pone.0069809
